# Supplementary material for: Electrochemical Characterization of Aromatic Molecules with 1,4-Diaza Groups for Flow Battery Applications
Source: Molecules. 2021 Apr 12;26(8):2227. doi: 10.3390/molecules26082227 (PMC8069459; doi:10.3390/molecules26082227)
Supplement: Supplementary file 1 [file molecules-26-02227-s001.pdf]

# Supporting Information

# Electrochemical Characterization of Aromatic Molecules with 1,4-Diaza Groups for Flow Battery Applications

Alexandros Pasadakis-Kavounis, Vanessa Baj, Johan Hjelm

Contents

**S1 Experimental Procedures** .......... **3**

**S1.1 General Experimental Considerations** .......... **3**

**S1.2 Synthesis** .......... **4**

S1.2.1 Synthesis of potassium pyrazine-2,3,5,6-tetracarboxylate **15** .......... 4

S1.2.2 Synthesis of sodium quinoxaline-2,3-diyldimethanesulfonate DSMeQUI **20** .......... 4

S1.2.3 Synthesis of 1,1′-(quinoxaline-2,3-diyl)bis(*N*,*N*,*N*-trymethylmethanaminium) DNMeQUI **21** .......... 5

**S1.3 NMR Experiments** .......... **6**

1.3.1 Comparison between 0.1 M solution of DSMeQUI **20** in 0.1 M KOH/0.9 M KCl and the same solution after 100 cycles .......... 6

1.3.2 Titration of QUI **6** in 0.1 M KOH/0.9 M KCl (in $H_2O$) .......... 7

1.3.3 Titration of DNMeQUI **21** in 0.1 M KOH/0.9 M KCl (in $H_2O$). .......... 8

**S1.4 Aggregation Constants** .......... **9**

**S1.5 Electrochemistry** .......... **11**

1.5.1 Cyclic voltammetry of DSMeQUI **20** with increasing concentrations. .......... 11

1.5.2 Charge/discharge cycles of DSMeQUI **20** in 0.1 M KOH/2 M KCl. .......... 12

1.5.3 Charge/discharge cycles of QUI **6** in 0.1 M KOH/1.55 M KCl. .......... 13

**S1.6 LC-MS analysis** .......... **14**

**S2 Equations** .......... **15**

S3 NMR Spectra ........................................................................................................ 16

References ........................................................................................................ 19

## S1. Experimental Procedures

### S1.1. General Experimental Considerations

All the starting materials, reagents, and solvents were purchased from commercial suppliers (Sigma-Aldrich, TCI Chemicals, Alfa Aesar, VWR International) and used without further purification. All the solvents were HPLC-grade. Thin-layer chromatography (TLC) was run on Merck aluminum plates pre-coated with 0.25 mm silica gel C-60 $F_{254}$. The plates were analyzed under UV light (254 nm). Eluent systems were expressed as volume ratios and specified for every $R_f$-value. Evaporation of the solvents was performed with a Buchi Rotavapor R-300 in vacuo at temperatures ranging between 25 and 60 °C. Traces of solvents were evaporated under reduced pressure by means of an oil pump.

$^1$H, $^{13}$C, and two-dimensional NMR spectra were recorded on a Magritek spectrometer, operating at 80 MHz ($^1$H) or 20 MHz ($^{13}$C) or on a Bruker Ascend spectrometer, operating at 400 MHz ($^1$H) or 100 MHz ($^{13}$C). Unless specified otherwise, all spectra were acquired at room temperature and referenced to the main internal solvent residue, which was water (4.79 ppm). Chemical shifts (δ) are listed in ppm and have uncertainties of ± 0.01 ppm for $^1$H, and ± 0.05 for $^{13}$C. Coupling constants (*J*) are quoted in Hertz. The following abbreviations are used for convenience in reporting the multiplicity for NMR resonances: s, singlet; d, doublet. Assignment of all $^1$H and $^{13}$C resonances was achieved using standard 2D NMR techniques: COSY, HSQC, and HMBC.

## S1.2. Synthesis

### S1.2.1. Synthesis of potassium pyrazine-2,3,5,6-tetracarboxylate 15

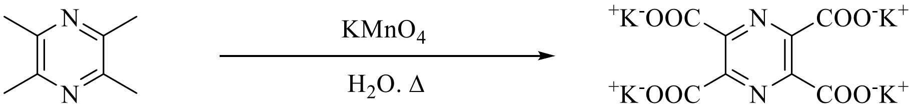


**Scheme S1.** Synthetic scheme for the synthesis of potassium pyrazine-2,3,5,6-tetracarboxylate **15** following the procedure in [26].

*Potassium pyrazine-2,3,5,6-tetracarboxylate (15)*

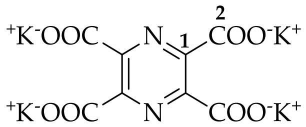

A solution of $KMnO_4$ (30.17 g, 0.19 mol, 13 eq.) in $H_2O$ (135 mL) was added dropwise to a pre-warmed (40 °C) suspension of pyrazine-2,3,5,6-tetramethyl (2.00 g, 0.015 mol, 1 eq.) in 0.45 M KOH (40 mL). The purple mixture was heated at reflux overnight. After cooling to room temperature, EtOH was added until the mixture became brown. The mixture was filtrated, the solid $MnO_2$ washed with $H_2O$, and the filtrate concentrated in vacuo to reduce the volume. A 10% solution of HCl was then added to induce the precipitation of the product. The solid was vacuum filtrated to yield the pure product (1.27 g) as a white solid.

Yield: 21%.

**$^{13}$C NMR** (10% KOH in $D_2O$) = δ(ppm): 172.1 (**C**2), 147.7 (**C**1).

### S1.2.2. Synthesis of sodium quinoxaline-2,3-diyldimethanesulfonate DSMeQUI 20

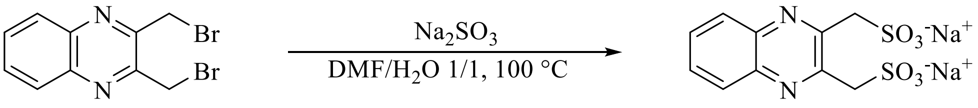


**Scheme S2.** Synthetic scheme for the synthesis of sodium quinoxaline-2,3-diyldimethanesulfonate DSMeQUI **20** following the procedure in [27].

*Sodium quinoxaline-2,3-diyldimethanesulfonate DSMeQUI (20)*

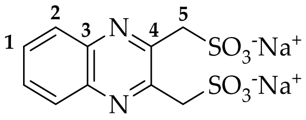

A solution of $Na_2SO_3$ (1.76 g, 0.014 mol, 2.2 eq.) in $H_2O$ (10 mL) was added to a solution of 2,3-bis(bromomethyl)quinoxaline (2.00 g,

0.006 mol, 1 eq.) in DMF (10 mL). The mixture was heated at reflux overnight. After cooling to room temperature, the solution was diluted with $H_2O$ (20 mL) and EtOH (12 mL), gravity filtered, and then washed with DCM (3 x 40 mL). The water layer was concentrated to dryness in vacuo to give a beige solid. The crude (3.45 g) was purified by one wash with MeOH (30 mL) to give the pure product (1.45 g) as a white solid.

Yield: 67%; $\mathbf{R}_f$ = 0.0 (heptane:EtOAc, 1:1).

$^1$**H NMR** ($D_2O$) = δ(ppm): 8.01 (2H, dd, *J* = 3.6 Hz and *J* = 6.5 Hz, **H**2), 7.81 (2H, dd, *J* = 3.4 Hz and *J* = 6.5 Hz, **H**1), 4.88 (4H, s, **H**5). $^{13}$**C NMR** ($D_2O$) = δ(ppm): 147.7 (**C**4), 140.7 (**C**3), 131.5 (**C**1), 127.9 (**C**2), 56.2 (**C**5).

### S1.2.3. Synthesis of 1,1′-(quinoxaline-2,3-diyl)bis(*N*,*N*,*N*-trymethylmethanaminium) DNMeQUI 21

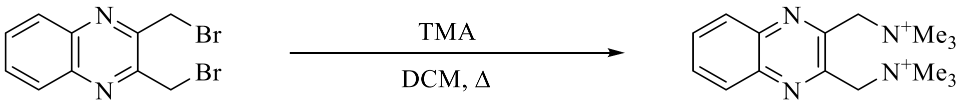


**Scheme S3.** Synthetic scheme for the synthesis of 1,1′-(quinoxaline-2,3-diyl)bis(*N*,*N*,*N*-trymethylmethanaminium) DNMeQUI **21** following the procedure in [28].

***1,1′-(quinoxaline-2,3-diyl)bis(N,N,N-trymethylmethanaminium) DNMeQUI (21)***

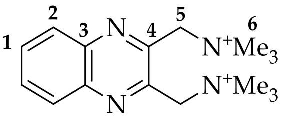


2,3-bis(bromomethyl)quinoxaline (2.01 g, 0.006 mol, 1 eq.) was dissolved in DCM (300 mL). Trimethylamine TMA (28.5 mL, 0.304 mol, 48 eq.) was added dropwise. The mixture was heated at reflux overnight. After cooling to room temperature, the solution was quenched with 100 mL of $H_2O$. The two phases were separated, and the water phase was washed with DCM (3 x 100 mL). The water layer was concentrated to dryness in vacuo to give a white solid. The crude product (5.91 g) was dissolved in 20 mL of $H_2O$ and then passed through 124 g of Amberlite® IRA-900 resin (chloride form) under vacuum washing the column with 200 mL of $H_2O$. The solvent was evaporated in vacuo to give a white solid purified by crystallization with acetone/2-propanol 1/6. A total of 353 mg of pure product as a white solid was obtained.

Yield: 17%; **R**$_f$ = 0.0 (hexane:EtOAc, 1:1).

**$^1$H NMR** ($D_2O$) = δ(ppm): 8.39-8.22 (2H, m, **H**2), 8.17-8.01 (2H, m, **H**1), 5.13 (4H, s, **H**5), 3.53 (18H, s, **H**6). **$^{13}$C NMR** ($D_2O$) = δ(ppm): 148.9 (**C**4), 140.6 (**C**3), 133.0 (**C**1), 128.9 (**C**2), 64.6 (**C**5), 54.3 (**C**6).

### S1.3. NMR Experiments

#### S1.3.1. Comparison between 0.1 M solution of DSMeQUI 20 in 0.1 M KOH/0.9 M KCl and the same solution after 100 cycles

DSMeQUI (**20**) (100 mM) was dissolved in 0.1 M KOH/0.9 M KCl and analyzed by $^1$H-NMR spectroscopy. The same solution was submitted to 100 charge–discharge cycles (paired with excess $K_4[Fe(CN)_6]$ in a flow battery system) and then re-analyzed in the same way.

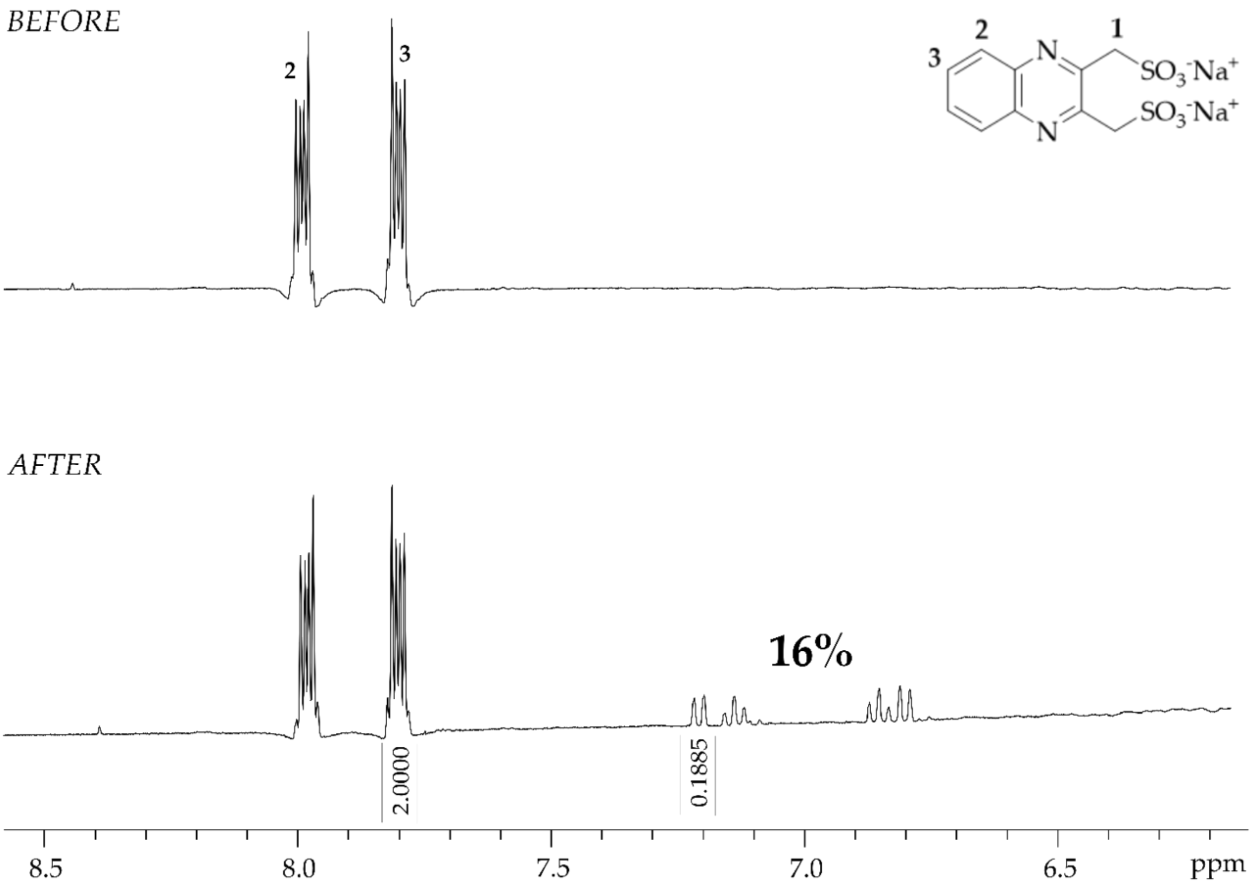


**Figure S1.** Aromatic region of the $^1$H-NMR spectra before and after 100 charge–discharge cycles. The spectra were recorded using a Bruker Ascend spectrometer, operating at 400 MHz ($^1$H).

### S1.3.2. Titration of QUI 6 in 0.1 M KOH/0.9 M KCl (in $H_2O$).

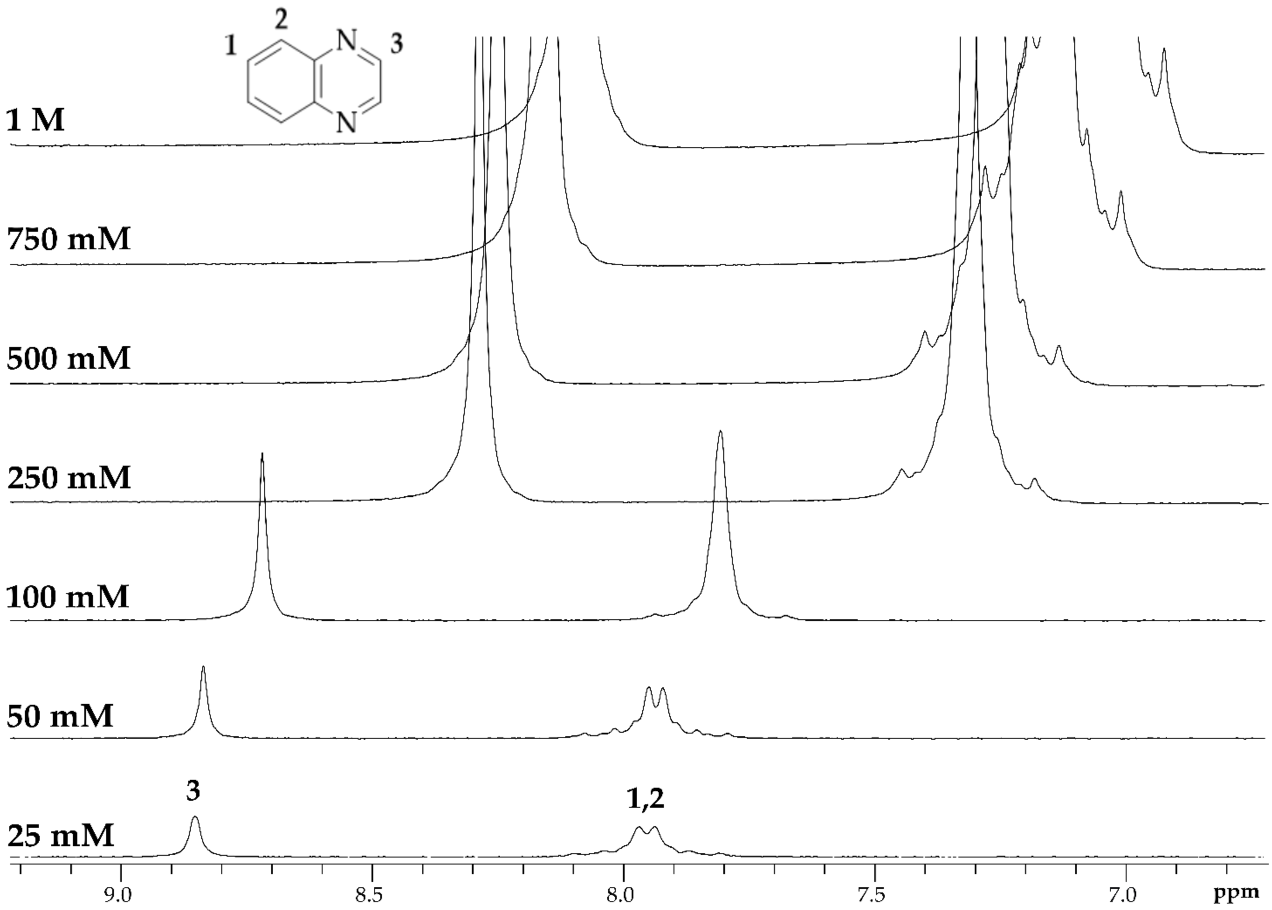


**Figure S2.** $^{1}$H-NMR spectra with increasing concentration of QUI **6**. The spectra were recorded using a Magritek spectrometer, operating at 80 MHz ($^{1}$H).

### S1.3.3. Titration of DNMeQUI 21 in 0.1 M KOH/0.9 M KCl (in $H_2O$).

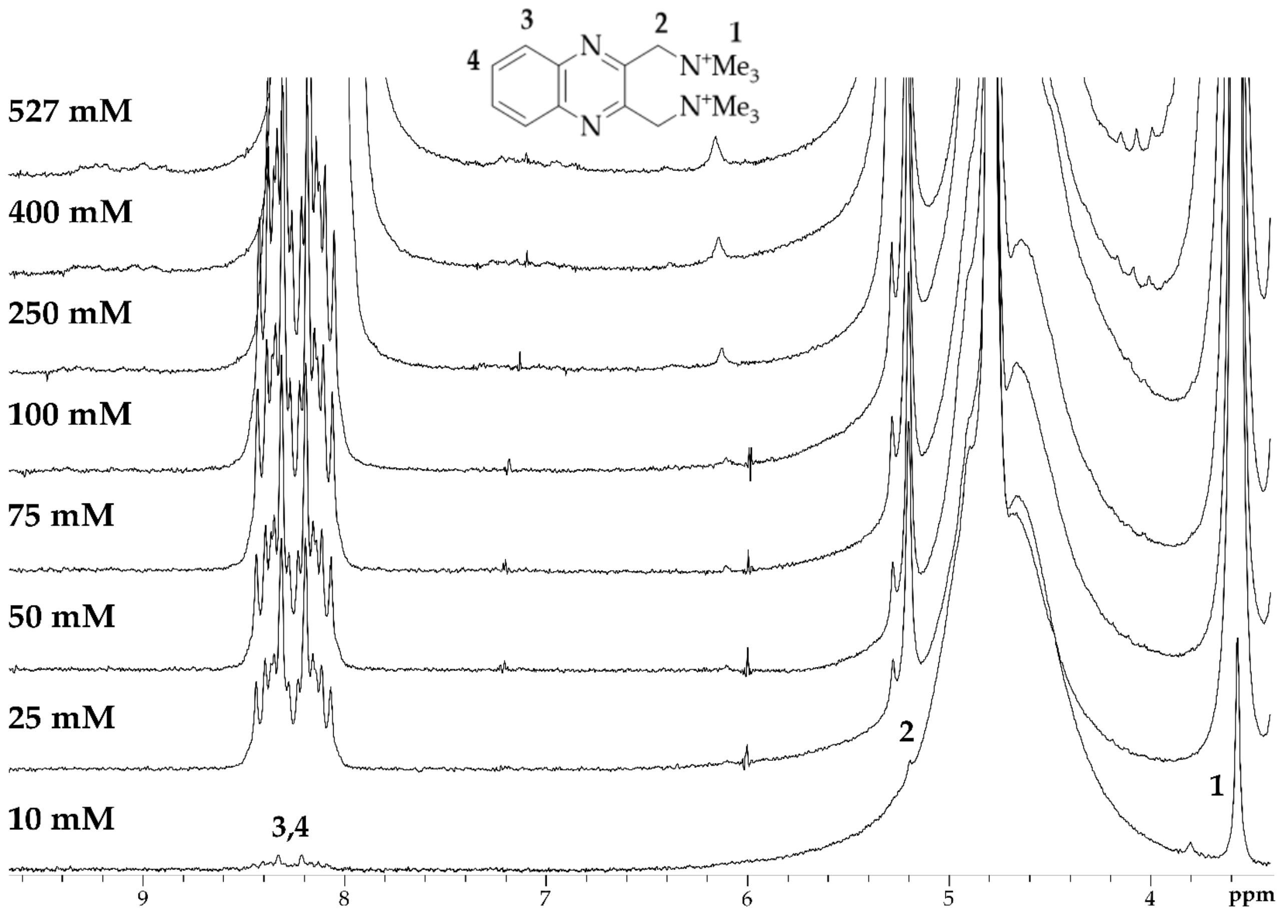


**Figure S3.** $^1$H-NMR spectra with increasing concentration of DNMeQUI **21**. The spectra were recorded using a Magritek spectrometer, operating at 80 MHz ($^1$H).

## S1.4. Aggregation Constants

**a) Hormann & Dreux model**

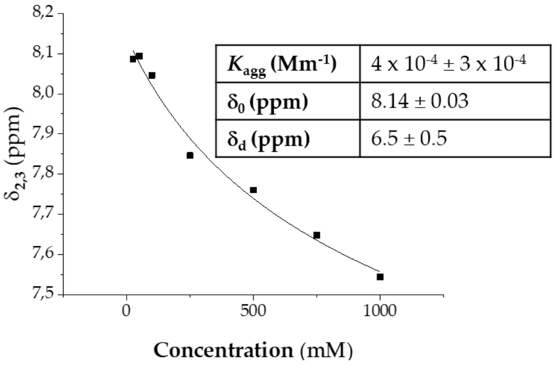


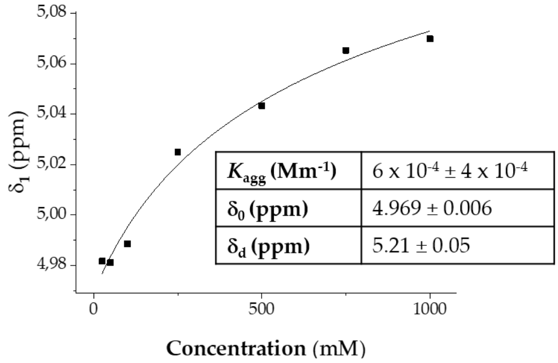


**b) Isodesmic model**

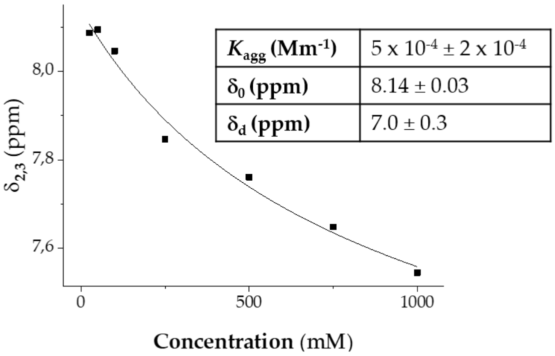


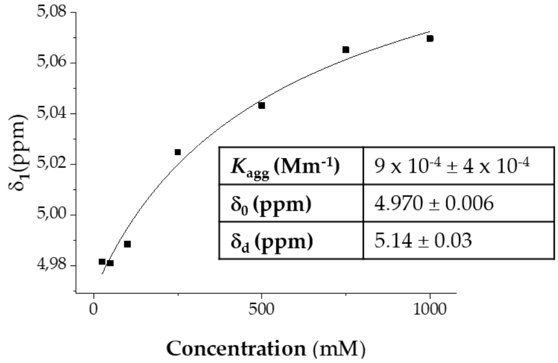


**Figure S4.** Non-linear least squares fitting of the chemical shifts of protons 2,3 ($\delta_{2,3}$) and proton 1 ($\delta_1$) of DMeSQUI **20** as a function of its total concentration in 0.1 M KOH/0.9 M KCl (**a**) according to the model of Hormann and Dreux, and (**b**) according to the isodesmic model.

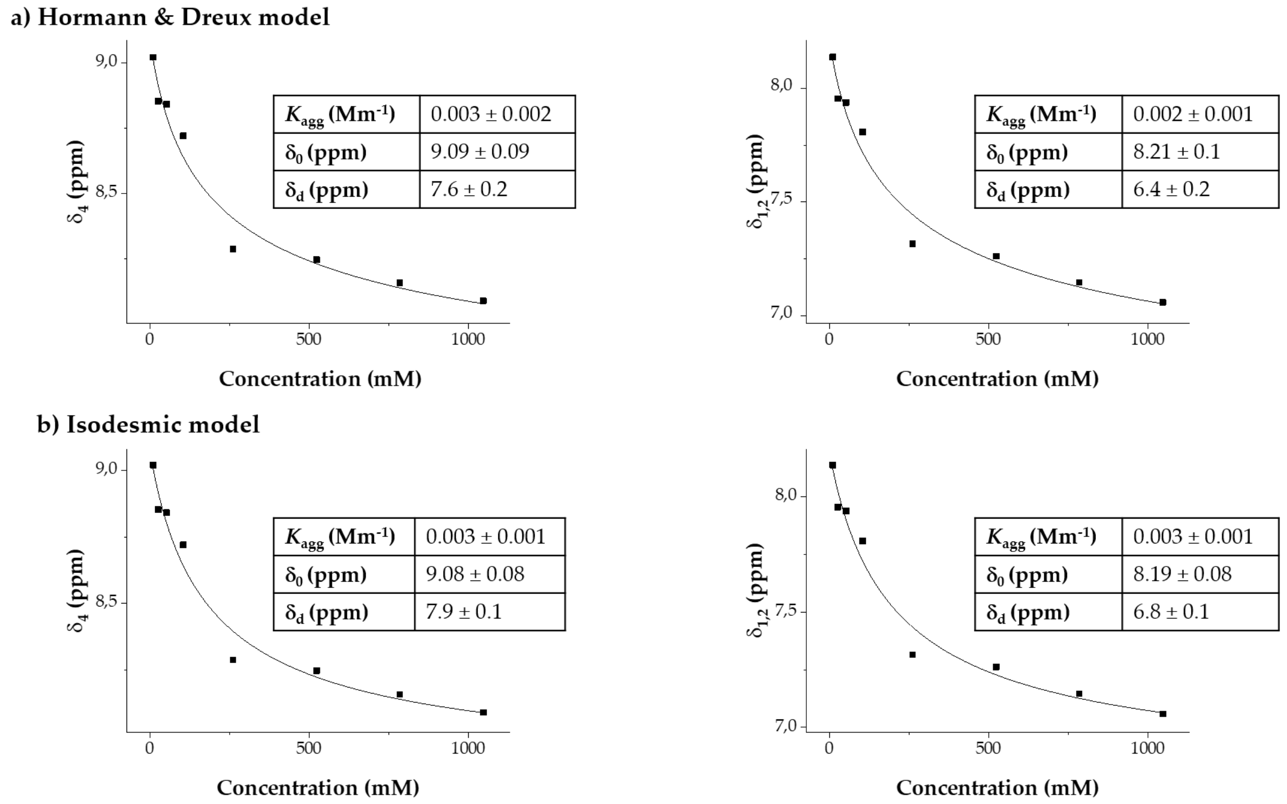


**Figure S5.** Non-linear least squares fitting of the chemical shifts of proton 4 ($\delta_4$) and protons 1,2 ($\delta_{1,2}$) of QUI **6** as a function of its total concentration in 0.1 M KOH/0.9 M KCl (**a**) according to the model of Hormann and Dreux, and (**b**) according to the isodesmic model.

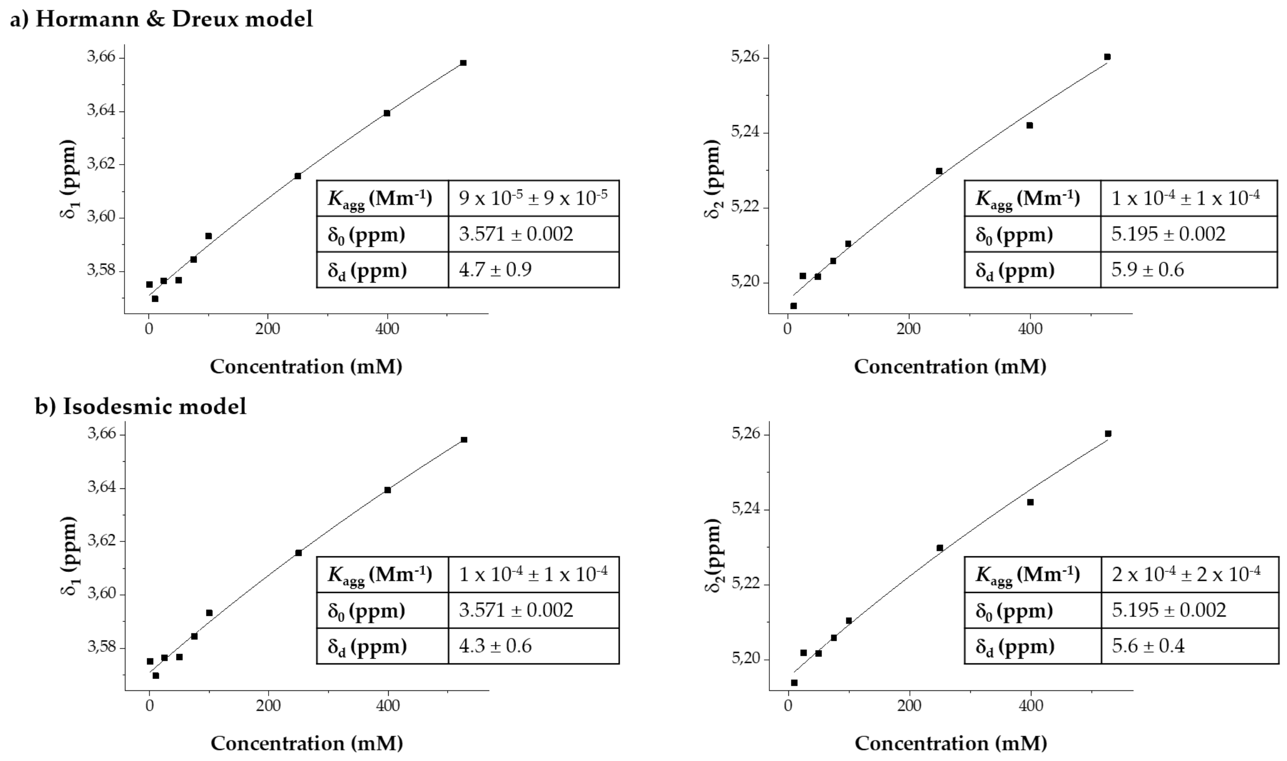


**Figure S6.** Non-linear least squares fitting of the chemical shifts of proton 1 ($\delta_1$) and proton 2 ($\delta_2$) of DNMeQUI **21** as a function of its total concentration in 0.1 M KOH/0.9 M KCl (**a**) according to the model of Hormann and Dreux, and (**b**) according to the isodesmic model.

## S1.5. Electrochemistry

### S1.5.1. Cyclic voltammetry of DSMeQUI 20 with increasing concentrations

Figure S7 displays cyclic voltammograms of DSMeQUI **20** in a range of concentrations from 1 to 250 mM. The background charging current has been removed and an iR correction has been made for each voltammogram. Impedance spectroscopy was used to determine the ohmic resistance of the cell in each case. The currents were normalized by the concentration. The inset scatter plot in Figure S7 indicates the cathodic peak current as a function of the concentration on a logarithmic scale. The electrolyte was 0.1 M KOH/0.9 M KCl for concentrations lower than 100 mM. For the 100 mM concentration, the KCl concentration was adjusted to 1.9 M, whereas at 250 mM, the concentration of KCl was adjusted to 3 M to reduce migration effects and IR drop. The pH of all solutions was 12.7–13.

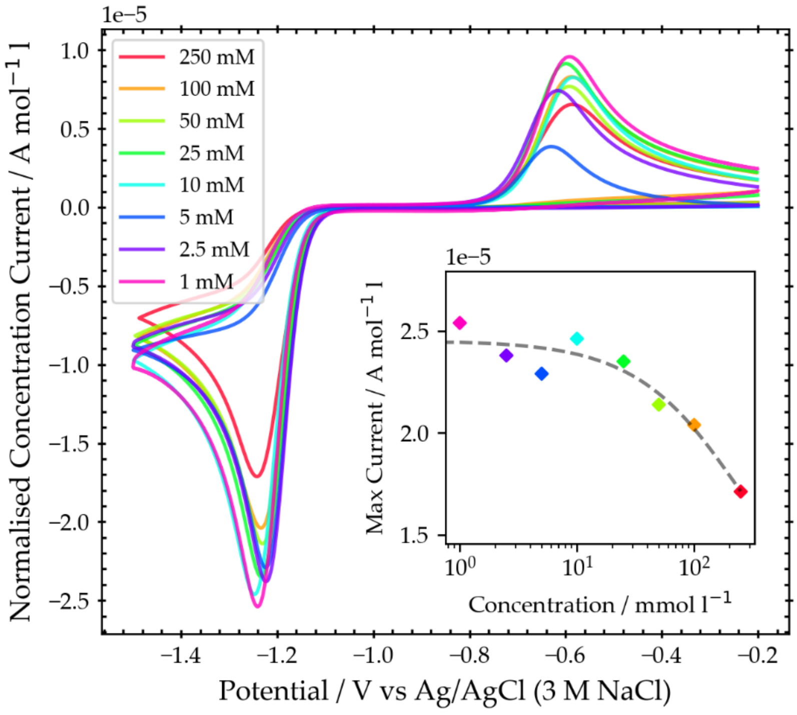


**Figure S7.** Cyclic voltammetry of DSMeQUI **20** at different concentrations at pH = 13. The voltammograms were recorded at a scan rate of 100 mV/s using a 3 mm diameter glassy carbon disk electrode. The inset shows the cathodic peak current (reduction of neutral quinoxaline) normalized to the concentration, as a function of the concentration.

### S1.5.2. Charge/discharge cycles of DSMeQUI 20 in 0.1 M KOH/2 M KCl

Charge–discharge cycles of 0.1 M DSMeQUI **20** dissolved in 0.1 M KOH/2 M KCl (negolyte) and 0.3 M $K_4(Fe(CN)_6)$/ 0.3 M $K_3(Fe(CN)_6)$ in 0.1 M KOH/0.9M KCl (posolyte) are shown in Figure S8c, while on the right, the capacity versus time and cycle is shown for the whole 110 cycles. The first cycle of was performed in constant current (0.1 A $cm^{-2}$)–constant voltage mode (CC–CV) to achieve exhaustive electrolysis of the capacity limiting DSMeQUI **20** negolyte. The CC–CV mode was repeated every 10 cycles. Initially, the current was constant until the potential reached an upper (1.6 V) or lower (0.1 V) cut-off value and then kept constant at the cut-off value until the current dropped below 10% of the initial (constant) current.

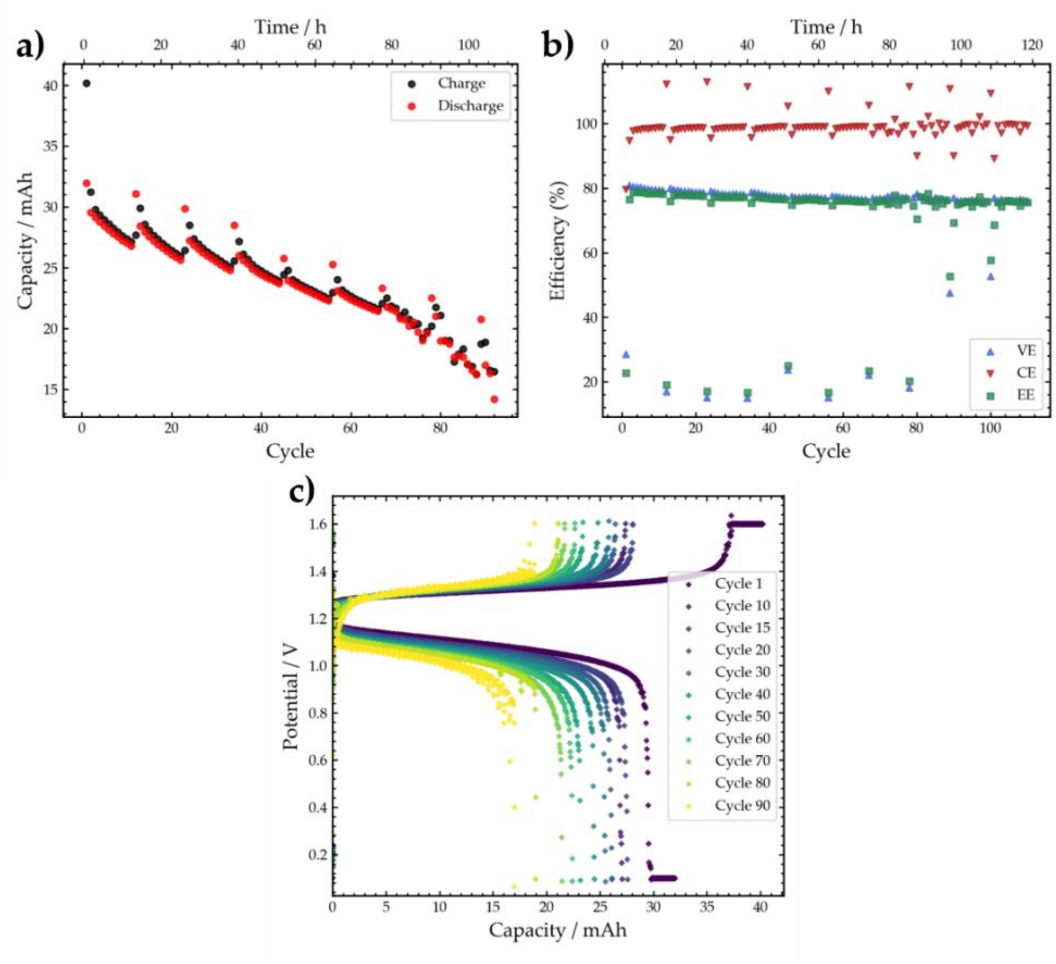


**Figure S8**. (**a**) Charge–discharge cycles vs cycles and time of 0.1M DSMeQUI **20** (10 mL) as analyte and 0.3 M $K_4(Fe(CN)_6)$/ 0.3 M $K_3(Fe(CN)_6)$ as catholyte circulated at a flow rate of 15 mL $min^{-1}$. The theoretical capacity of the limiting side was 53.6 mAh. The theoretical capacity of the positive side was 80.4 mAh. Carbon cloth (hydrophilic plain weave and 400 μm thick carbon cloth from ELAT) was used as electrodes with a mPBI (10

micron) membrane as the separator. The cell was constructed using Poco graphite blocks (Fuel Cell Technologies) with serpentine flow fields. The geometric electrode area was 5 $cm^2$, and the cell was sealed using Viton gaskets. (**b**) The corresponding graph voltaic efficiency (VE), coulombic efficiency (CE), and energy efficiency (EE). (**c**) Charge–discharge curves of different cycles.

### S1.5.3. Charge/discharge cycles of QUI 6 in 0.1 M KOH/1.55 M KCl.

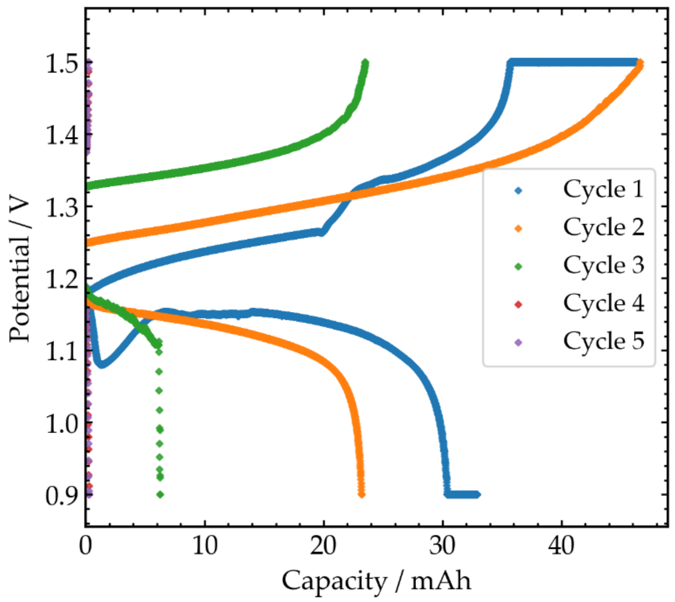


**Figure S9**. Charge–discharge cycles of 0.1M QUI **6** (10 mL) in 0.1 M KOH/1.55 M KCl as anolyte and 0.3 M $K_4(Fe(CN)_6)$ as catholyte circulated at a flow rate of 25 mL $min^{-1}$. The theoretical capacity of the limiting side was 80.4 mAh. The theoretical capacity of the positive side was 120.6 mAh. Carbon cloth (hydrophilic plain weave and 400 μm thick carbon cloth from ELAT) was used as electrodes with a Nafion 212 membrane as the separator. The cell was constructed using Poco graphite blocks (Fuel Cell Technologies) with serpentine flow fields. The geometric electrode area was 5 $cm^2$, and the cell was sealed using Viton gaskets.

## S1.6. LC–MS analysis

A 0.1 M QUI **6** solution in 0.1 M KOH/0.9 M KCl was inserted in a flow battery system as the negolyte paired with an excess of 0.3 M $K_4(Fe(CN)_6)$ as posolyte. Carbon cloth (hydrophilic plain weave and 400 μm thick carbon cloth from ELAT) was used as electrodes with a Nafion 212 membrane as the separator. The cell was constructed using Poco graphite blocks (Fuel Cell Technologies) with serpentine flow fields. The geometric electrode area was 5 $cm^2$, and the cell was sealed using Viton gaskets. After 5 cycles, the solution was analyzed by LC–MS, as shown in Figure S10. The capacity after 5 cycles had fallen to 0.

For the LC–MS trace, the following analysis method was run:

Solvent A: 0.1% TFA in MilliQ Water

Solvent B: 0.1% TFA in Acetonitrile

Gradient:

| Time / min | % Solution A | % Solution B |
|---|---|---|
| 0.00 | 95 | 5 |
| 4.00 | 0.0 | 100 |
| 4.50 | 0 | 100 |
| 5.00 | 95 | 5 |

A UV detector with an electron spray (ES) Lamp was used in the positive mode. The selected wavelengths ranged between 210 and 500 nm.

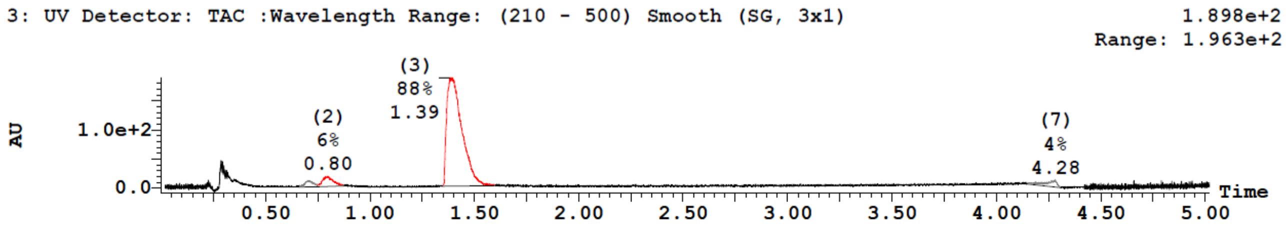


**Figure S10**. LC–MS trace of QUI (**6**) after 5 cycles in a flow battery test leading to failure of the battery (0% capacity). The two red peaks are the ones of interest. The biggest one at 1.39 min corresponds to a mass of 130.97 g $mol^{-1}$, which is QUI (**6**). The small red peak at 0.80 min has instead a mass of 146.86 g $mol^{-1}$

indicating that it is molecule **8a** (quinoxalin-2-ol)**.** The small peak at 0.62 min corresponded instead to a mass of 134.84, which matched the mass of the fully reduced form of quinoxaline (1,2,3,4-tetrahydroquinoxaline, molecule **10b**). The instrument method was only qualitative with respect to the amounts of the compounds, as wide band UV–Vis detection (210–250 nm) was used, and the individual components' extinction coefficients are not established. Therefore the peak areas only qualitatively indicate the amount of each compound and cannot be taken as mole fractions. Assuming the extinction coefficients of the degradation products (**8a, 9a**, **10b**) are not dramatically altered from that of quinoxaline (**6**), then quinoxaline was the majority component after test, even though the capacity had faded to nearly 0%.

## S2. Equations

The equation of the Horman and Dreux model is [25]

$$\delta_i = \delta_0 - \left[\left(1 + \frac{1}{4c_i K_{agg}}\right) - \sqrt{\left(1 + \frac{1}{4c_i K_{agg}}\right)^2 - 1}\,\right](\delta_0 - \delta_d)$$

where:

$\delta_i$: is the measured chemical shift at a concentration $c_i$; **OBSERVED VALUE**

$\delta_0$: is the limiting chemical shift of the monomer;

$\delta_d$: is the limiting chemical shift of the dimer; **UNKNOWN, FITTING PARAMETER**

$c_i$: is the concentration; **KNOWN VALUE**

$K_{agg}$: is the constant of aggregation. **UNKNOWN, FITTING PARAMETER**

$\delta_0$ is theoretically a known value, but a small percentage of dimer will always be present; therefore, it was treated initially as an unknown, such as $\delta_d$ and $K_{agg}$.

The equation of the isodesmic model is [23]

$$\delta_i = \delta_0 + \frac{4(\delta_d - \delta_0)K_{agg}c_i\left(2 - \frac{4K_{agg}c_i}{\left(1 + \sqrt{1 + 4K_{agg}c_i}\right)^2}\right)}{\left(1 + \sqrt{1 + 4K_{agg}c_i}\right)^2}$$

where:

$\delta_i$: is the measured chemical shift at a concentration $c_i$; **OBSERVED VALUE**

$\delta_0$: is the limiting chemical shift of the monomer;

$\delta_d$: is the limiting chemical shift of the dimer; **UNKNOWN, FITTING PARAMETER**

$c_i$: is the concentration; **KNOWN VALUE**

$K_{agg}$: is the constant of aggregation. **UNKNOWN, FITTING PARAMETER**

$\delta_0$ is theoretically a known value, but a small percentage of dimer will always be present; therefore, it was treated initially as an unknown parameter, such as $\delta_d$ and $K_{agg}$.

## S3. NMR Spectra

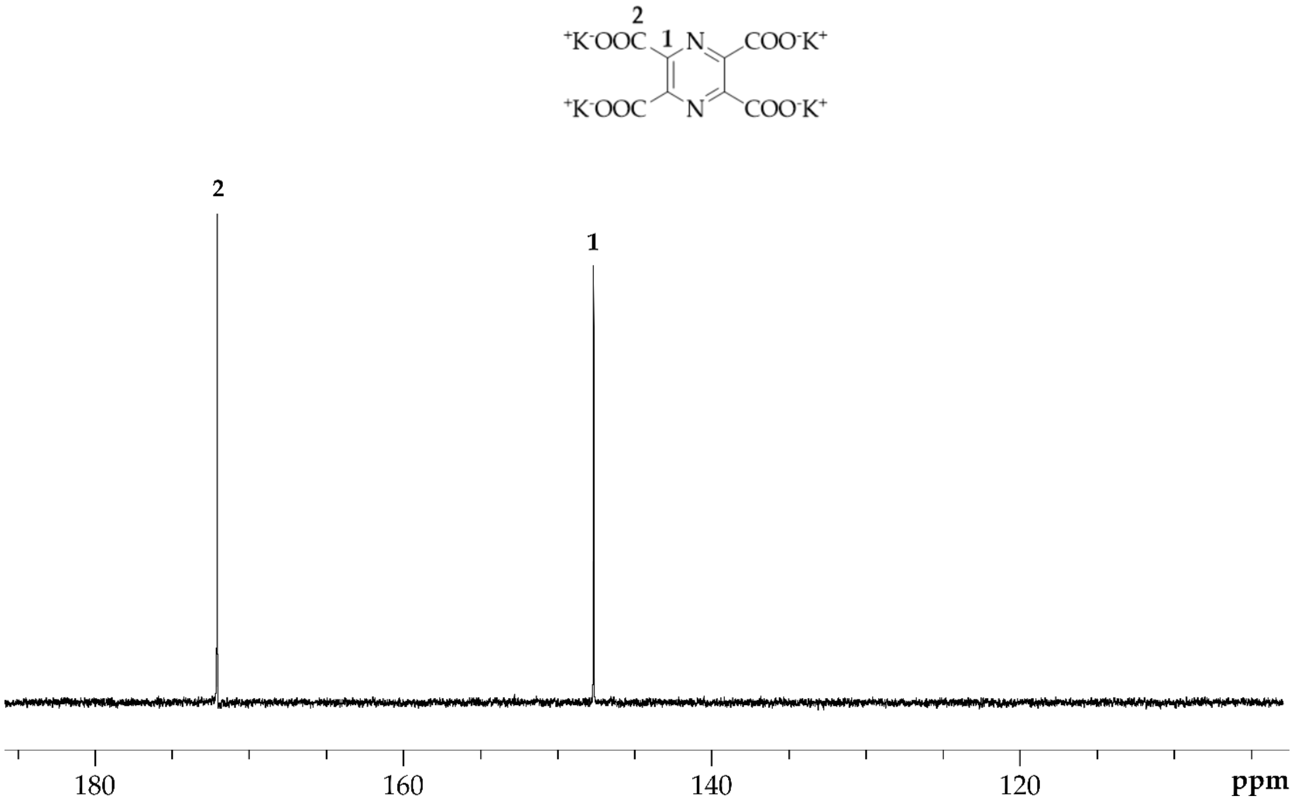


$^{13}$C NMR spectrum of compound **15**. The spectra was recorded using a Bruker Ascend spectrometer operating at 100 MHz ($^{13}$C).

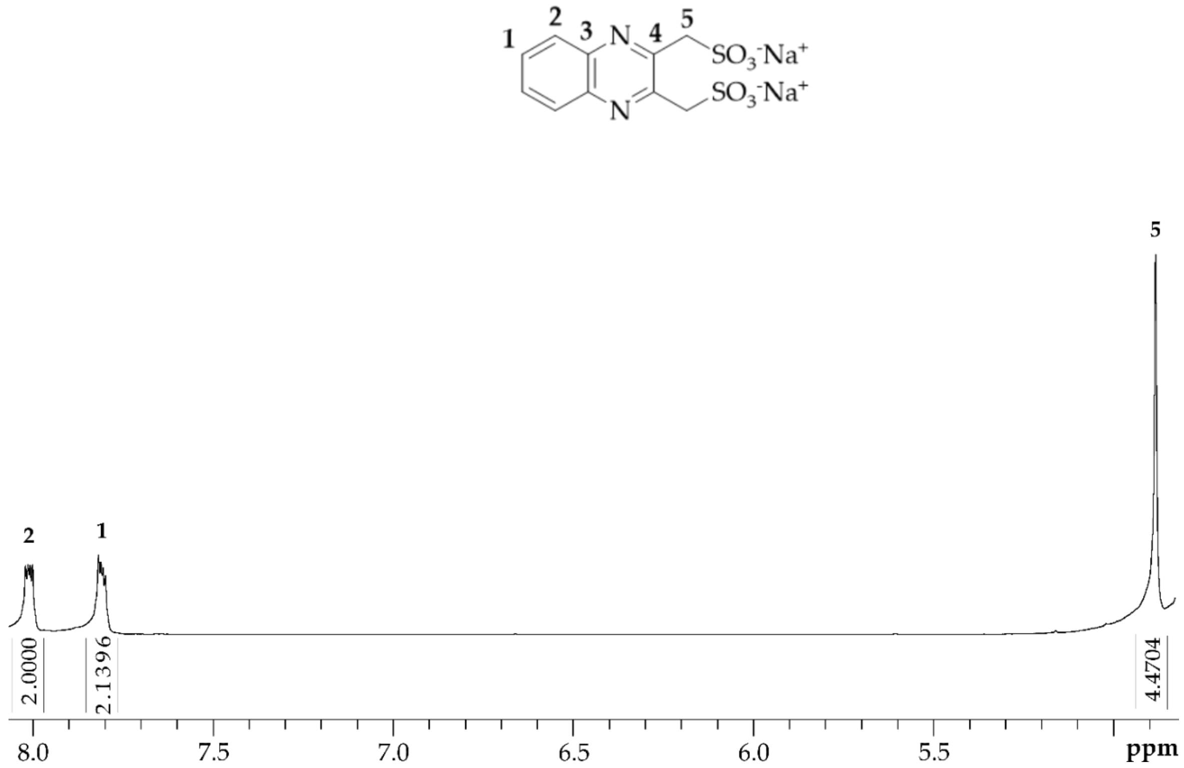


$^{1}H$ NMR spectrum of compound **20**. The spectra was recorded using a Bruker Ascend spectrometer operating at 400 MHz ($^{1}H$).

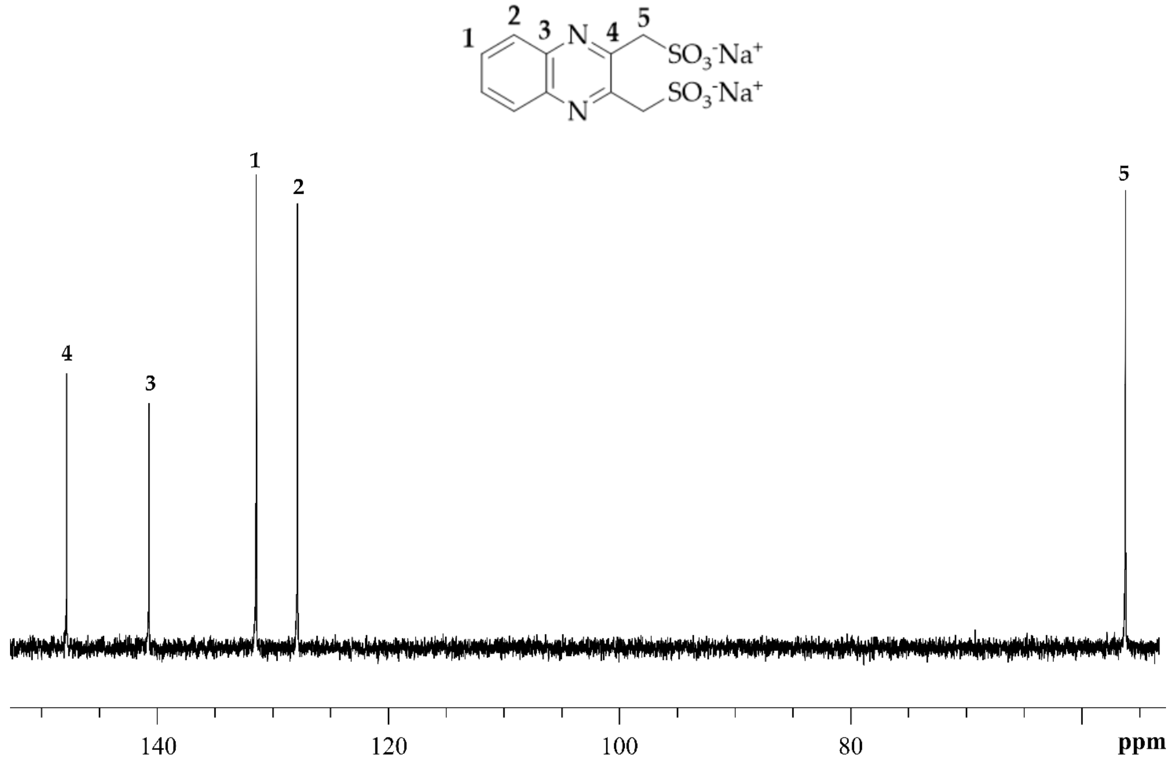


$^{13}C$ NMR spectrum of compound **20**. The spectra was recorded using a Bruker Ascend spectrometer operating at 100 MHz ($^{13}C$).

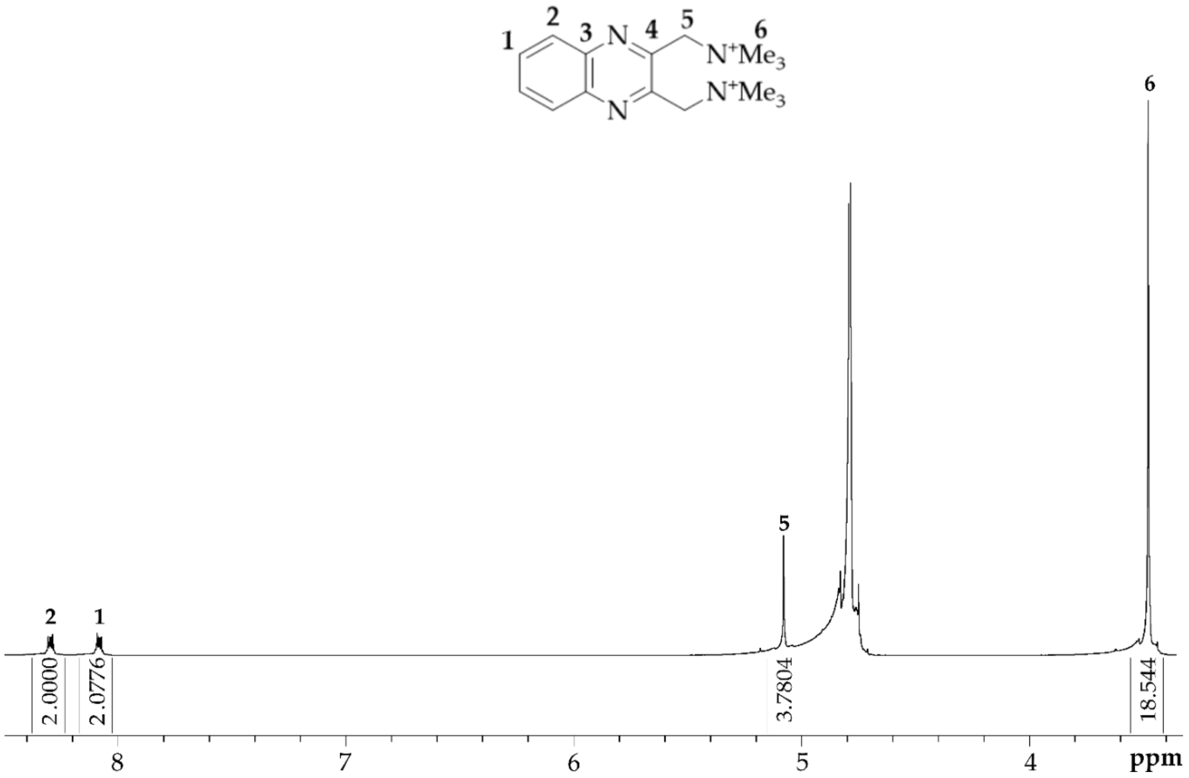


$^{1}$H NMR spectrum of compound **21**. The spectra was recorded using a Bruker Ascend spectrometer operating at 400 MHz ($^{1}$H).

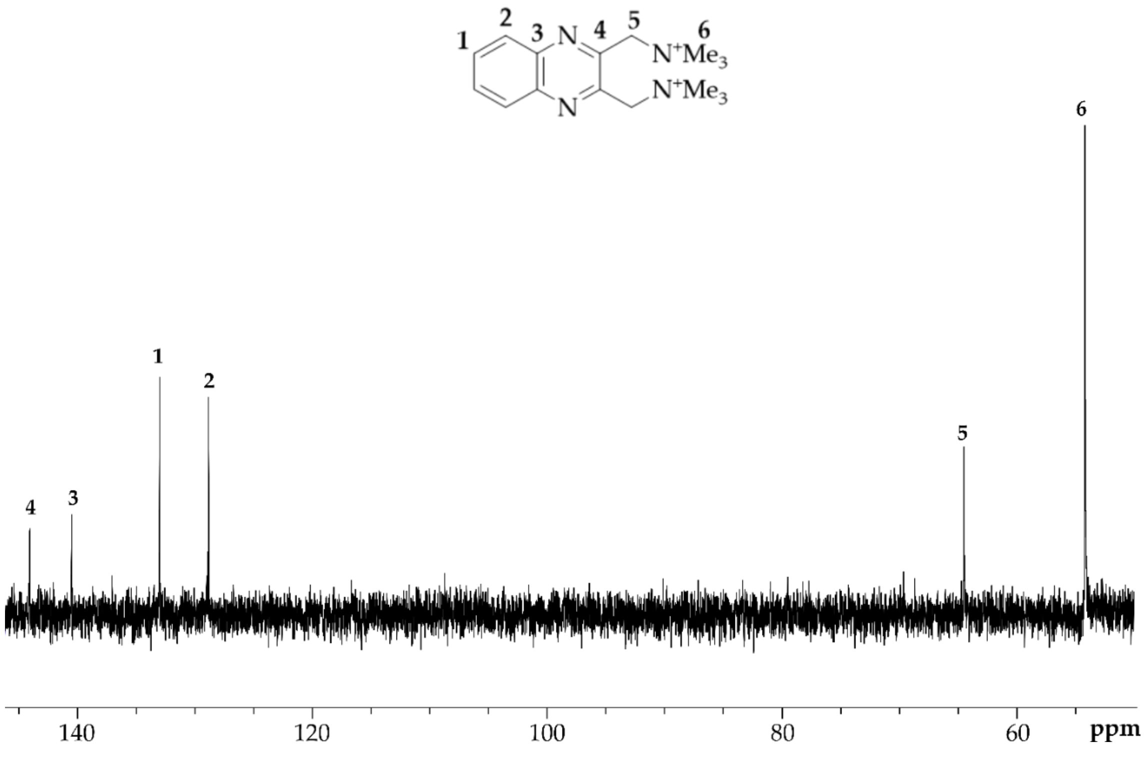


$^{13}$C NMR spectrum of compound **21**. The spectra was recorded using a Bruker Ascend spectrometer operating at 100 MHz ($^{13}$C).
